# The Value of the First Clinical Impression as Assessed by 18 Observations in Patients Presenting to the Emergency Department

**DOI:** 10.3390/jcm12020724

**Published:** 2023-01-16

**Authors:** Thomas Tschoellitsch, Stefan Krummenacker, Martin W. Dünser, Roland Stöger, Jens Meier

**Affiliations:** 1 Department of Anesthesiology and Critical Care Medicine, Kepler University Hospital, Johannes Kepler University Linz, 4020 Linz, Austria; 2 Kepler University Hospital, Johannes Kepler University Linz, 4020 Linz, Austria; 3 Praxis für Allgemein- und Familienmedizin, 4262 Leopoldschlag, Austria

**Keywords:** first clinical impression, prediction, triage, emergency medicine, emergency department, urgency, hospital admission, machine learning, artificial intelligence

## Abstract

The first clinical impression of emergency patients conveys a myriad of information that has been incompletely elucidated. In this prospective, observational study, the value of the first clinical impression, assessed by 18 observations, to predict the need for timely medical attention, the need for hospital admission, and in-hospital mortality in 1506 adult patients presenting to the triage desk of an emergency department was determined. Machine learning models were used for statistical analysis. The first clinical impression could predict the need for timely medical attention [area under the receiver operating characteristic curve (AUC ROC), 0.73; *p =* 0.01] and hospital admission (AUC ROC, 0.8; *p =* 0.004), but not in-hospital mortality (AUC ROC, 0.72; *p =* 0.13). The five most important features informing the prediction models were age, ability to walk, admission by emergency medical services, lying on a stretcher, breathing pattern, and bringing a suitcase. The inability to walk at triage presentation was highly predictive of both the need for timely medical attention (*p <* 0.001) and the need for hospital admission (*p <* 0.001). In conclusion, the first clinical impression of emergency patients presenting to the triage desk can predict the need for timely medical attention and hospital admission. Important components of the first clinical impression were identified.

## 1. Introduction

The first clinical impression of emergency patients conveys a myriad of information. Experienced clinicians recognize and correctly interpret the patient’s general appearance (e.g., skin color of the face, body position, ability to walk), subtle clinical signs (e.g., breathing pattern, the position of the patient’s hand), as well as acoustic (e.g., changes of the voice) and olfactory (e.g., urine or alcohol odor) clues within a few seconds of meeting an emergency patient. Despite its practical importance, the value of the first clinical impression in the assessment of emergency patients has not yet been scientifically elucidated. So far, only a handful of studies have shown that by using clinical intuition, emergency department staff can predict the need for hospital admission [1,2,3], presence of sepsis [4,5], disease severity, and the risk of short-term mortality [6,7,8]. However, in some of these studies, clinical intuition was also based on vital parameter readings [4], and none of these analyses established which clinical signs and indicators informed the clinicians’ intuition. Knowing what signs to look for and how to correctly interpret them would not only allow for better evaluation of emergency cases, but also for the structured education of young clinicians in good patient assessment.

In this clinical study, we determined the value of the first clinical impression, assessed by 18 observations, to predict the need for timely medical attention, the need for hospital admission, and in-hospital mortality in adult patients presenting to the triage desk of an emergency department.

## 2. Materials and Methods

The analysis was designed as an explorative, prospective, observational, single-center cohort study. During ten randomly chosen days from November 2019 until January 2020, it was conducted at the emergency department of the Kepler University Hospital, a tertiary university teaching hospital with 1830 beds. The emergency department serves all adult patients with emergency or acute conditions except for those following trauma and patients with obstetrical, psychiatric, or ophthalmologic emergencies. The study protocol was reviewed and approved by the ethics committee of Johannes Kepler University (1175/2019). In view of the fact that written informed consent before study enrolment would have highly likely influenced the study results by changing the patients’ behavior when entering the emergency department, no study-related interventions were made, and only anonymized data were documented, and written informed consent was waived.

### 2.1. Study Patients

All patients presenting to the triage area of the emergency department were eligible for study enrollment. Patients < 18 years were excluded. In addition, patients who were referred to the emergency department by emergency medical services and were in a critical or life-threatening condition were not evaluated in the triage area but were directly admitted to the resuscitation bay. Similarly, patients who were referred by emergency medical services and had a high clinical suspicion of a stroke or ST-elevation myocardial infarction were directly admitted to one of the emergency department’s acute care areas.

### 2.2. Data Collection

Data collection took place in the emergency department’s triage area, which is a large room with two triage desks, each with a chair in front of the desk. The triage area is separated by a wall that ensures patient confidentiality but also allows to oversee both triage desks at its end. Study-related data were only gathered from the moment the patient entered the room until medical history taking started. Triage was performed by specially trained triage nurses with the use of a computer-based Manchester Triage System application. On workdays between 7 a.m. and 7 p.m., study data were collected in real time using an electronic case report form on a hand-held device. To eliminate inter-rater variability, all first clinical impression-related study data were collected by one final-year medical student. Before data collection started, the student had worked in the emergency department as a physician assistant for 6 weeks. During this time, specific emphasis was put on training the student to recognize clinical signs and indicators as screened for in the case report form.

The first clinical impression was assessed by collecting 18 clinical observations. In view of the lack of objective criteria to describe components of the first clinical impression, these 18 observations were defined by a group of experienced physicians and nurses of the study hospital. The case report form was anonymized and included the admission number of the patient, time and mode of admission, ability to walk at triage presentation (assessed by the observation of how the patient entered the room and approached the triage desk), body posture when seated or lying on a stretcher, order of sheets on the stretcher, facial expression, skin color of the face, breathing pattern, mental impression, presence of a vomitus bag, type of clothes and shoes, hygienic state, presence of a suitcase or chaperone, and conversation details (before medical history taking started). Using the admission number on the electronic case report forms, another researcher extracted the following data from the electronic data system of the emergency department: age, sex, vital parameters measured in the triage area, management priority as defined by the Manchester Triage System, need for timely medical attention, need for hospital admission, need for intensive care unit admission, and in-hospital mortality. All data were merged into one electronic database, which was locked following the completion of patient recruitment.

### 2.3. Definitions

Study patients were categorized as being unable to walk when they were admitted to the emergency department triage area, either in an EMS chair or on an EMS stretcher. The need for timely medical attention was defined in accordance with the Manchester Triage System categories of red and orange. These categories are referred to as “immediate” and “very urgent” with a maximum waiting time until medical attention of 0 and 10 min, respectively.

### 2.4. Study Endpoints

The primary endpoint of this study was to determine the value of the first clinical impression to predict the need for timely medical attention, the need for hospital admission, and in-hospital mortality. As secondary study endpoints, we sought to identify the five most important features contributing to the prediction of each of the primary study endpoints (if significant).

### 2.5. Statistical Analysis

In view of the scarce published literature on this topic and the explorative character of our study, no sample size calculation could be performed. We assumed that enrolment of a convenience sample of 1500 patients would be sufficient to produce clinically meaningful answers to the study questions.

Following the locking of the database and plausibility control of all entered values, statistical analyses were performed using the R software package (R version 4.1.2; https://www.R-project.org/ (accessed on 1 October 2022). Descriptive statistical methods were applied to report demographic, clinical, and outcome data. To determine the value of the first clinical impression (including all 18 observations and the time of admission) to predict the need for timely medical attention, the need for hospital admission, and in-hospital mortality, we used these endpoints as binary variables and applied automated machine learning methods [9]. The package (AutoML 3.36.1.5; H_2_O.ai (accessed on 1 October 2022), Mountain View, CA, USA) used includes the process of automating tasks in the machine learning pipeline, such as data preprocessing, hyperparameter tuning, model selection, and evaluation. H_2_O AutoML trains and cross-validates the following models: three pre-specified XGBoost gradient boosting machine models, a fixed grid of generalized linear models, a default random forest, five pre-specified H_2_O gradient boosting machine models, a near-default deep neural net, an extremely randomized forest, a random grid of XGBoost gradient boosting machine models, a random grid of H_2_O gradient boosting machine models, a random grid of deep neural nets, a stacked ensemble of all the models trained above, as well as a “best of family” stacked ensemble that contains the best-performing model for each algorithm class. After the models had been trained, the model performance was compared using log loss and root mean squared error. The best model was then chosen for the prediction. Training of the model was conducted after a random 80%/20% (training dataset/validation dataset) split of the data on the training data set, whereas validation was performed on the validation data set. In order to determine the feature importance of each single feature, we used the Boruta package for R (version 7.0.0). For validation, cross-tabulation analyses were applied to define the sensitivity, specificity, and positive and negative predictive values of selected single features.

Data are presented as median values with interquartile ranges or absolute values with percentages. *p*-values < 0.05 were considered to indicate statistical significance.

## 3. Results

**Figure 1:**
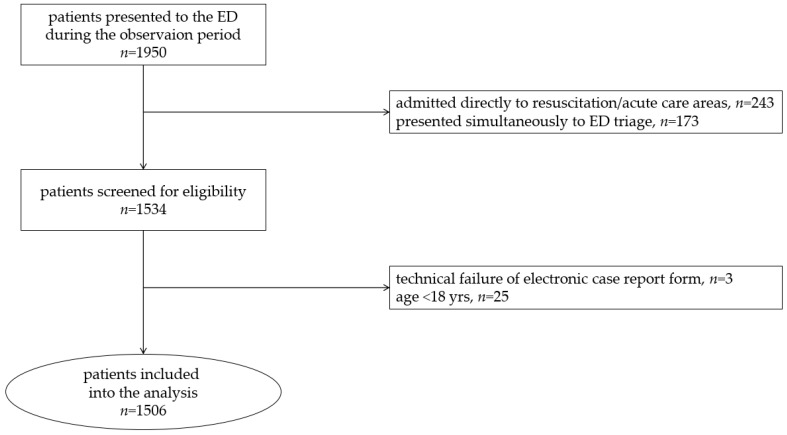
CONSORT flow diagram. ED, emergency department.

During the observation period, 1950 patients presented to the emergency department, of whom 1534 were screened for eligibility and 1506 were included in the analysis (Figure 1). The characteristics of the study population are summarized in Table 1.

The first clinical impression could predict the need for timely medical attention with an area under the receiver operating characteristic curve (AUC-ROC) of 0.73, a sensitivity of 53.1%, a specificity of 77.3%, a positive predictive value of 38.6%, a negative predictive value of 86%, and an accuracy of 72.2% (*p =* 0.01). Similarly, the first clinical impression could predict the need for hospital admission with an AUC ROC of 0.8, a sensitivity of 77.1%, a specificity of 70.7%, a positive predictive value of 62.8%, a negative predictive value of 82.8%, and an accuracy of 73.2% (CI95%, 67.8–78.1%) (*p =* 0.004). The first clinical impression could not predict in-hospital mortality (AUC ROC, 0.72; sensitivity, 0%; specificity, 100%; positive predictive value, 0%; negative predictive value, 98.7%; accuracy, 98.7%) (*p =* 0.13).

**Figure 2:**
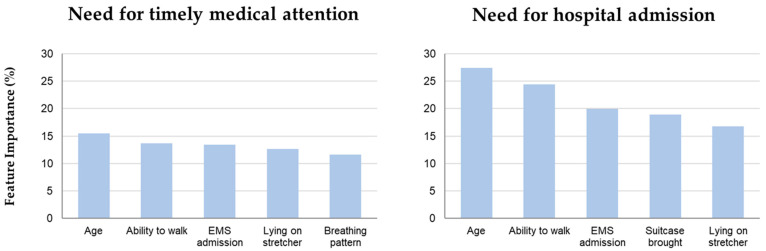
Feature importance of automated machine learning models to predict the need for timely medical attention and the need for hospital admission. EMS, emergency medical services.

**Figure 3:**
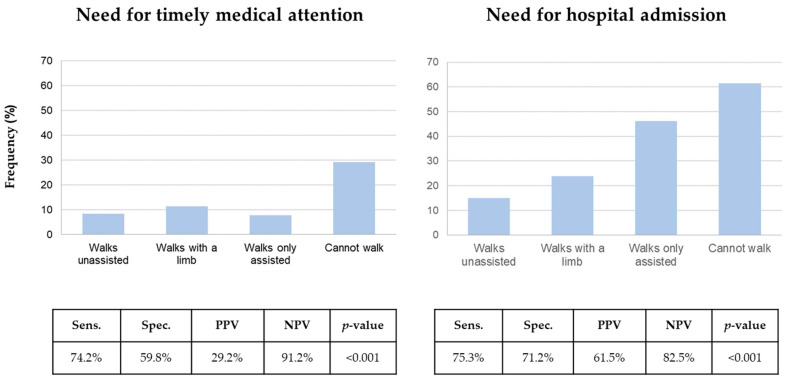
Frequencies of the need for timely medical attention and hospital admission depending on the ability to walk as well as the predictive value of the inability to walk at triage presentation. NPV, negative predictive value; PPV, positive predictive value; Sens., sensitivity; Spec., specificity.

The five most important features informing the models to predict the need for timely medical attention and the need for hospital admission are presented in Figure 2. Following age, the ability to walk at triage presentation was the most important clinical feature in predicting whether a patient required timely medical attention or hospital admission. Frequencies of the need for timely medical attention and hospital admission depending on the ability to walk as well as the predictive value of the inability to walk at triage presentation are displayed in Figure 3.

## 4. Discussion

In this prospective, observational study, we found that the first clinical impression of patients presenting to the triage desk in an emergency department could reliably predict the need for timely medical attention and the need for hospital admission, but not in-hospital mortality. Important features informing the prediction models were age, ability to walk, admission by emergency medical services, lying on a stretcher, breathing pattern, and bringing a suitcase. The inability to walk at triage presentation was highly predictive of both the need for timely medical attention and the need for hospital admission.

Due to center-specific admission pathways for critically ill patients and those with time-critical conditions such as ST-elevation myocardial infarction and stroke, our population largely included non-critically ill patients. This is important to remember when interpreting the study results and comparing our results to those of other authors. We deliberately chose one final-year medical student to collect data on the first clinical impression. Thus, we avoided any inter-rater bias and made sure that the clinical experience of the researcher did not bias the recognition and interpretation of components of the first clinical impression. Another methodological aspect of this study requiring discussion is the statistical analysis. We applied machine learning methods in an attempt to simulate the human brain’s ability to simultaneously gather and, most importantly, process a multitude of non-linear information [10,11,12].

The first clinical impression could reliably predict the need for timely medical attention in this population. Our study is the first to highlight this specific value of the first clinical impression of emergency patients. We used the two highest management priorities of the Manchester Triage System to define the need for timely medical attention. Although validation testing of the Manchester Triage System in a general emergency department has shown good results [13], it has also been criticized for potential under-triage at higher urgency levels [14]. The finding that the first clinical impression predicts the need for hospital admission with high accuracy confirms the results of previous research. Two prospective, observational studies from Switzerland using a similar set-up found that clinical intuition ratings of emergency physicians achieved comparable values to predict the need for hospital admission as observed in our analysis [1,2]. Similarly, studies conducted in emergency departments in the Netherlands and Canada reported that the intuition of clinicians, informed by the first clinical impression before triage, could predict the need for hospitalization [3,8]. The result that the first clinical impression was not predictive of in-hospital mortality in our cohort was surprising but is likely to be due to the fact that the number of patients dying during hospital admission was very low (*n* = 10, 0.7%), rendering the analysis underpowered to detect a significant association using machine learning methods. This is in contrast to other studies, which reported that the first clinical impression could also predict the risk of death [3,8].

An important feature of our study is that we did not use intuition to interpret the first clinical impression but evaluated 18 specific components of the first clinical impression. This allowed us to determine which observations of the first clinical impression might be especially important to screen for when evaluating an emergency patient at triage. Our results suggested six components of particular relevance. Apart from age, the ability to walk at triage presentation was the most important clinical feature informing both prediction models. Being unable to walk at triage presentation was highly predictive of the need for timely medical attention and hospital admission. This appears physiologically sound and might not only reflect the degree of frailty [15] but also the extent to which the patient’s physiologic reserve has been compromised by the acute condition. Similarly, the inability to walk at hospital admission proved to be an accurate predictor of the risk of in-hospital death in three African studies [16,17,18].

Certain limitations need to be taken into account when interpreting the results of our study. First, 18 components of the first clinical impression were selected based on clinical experience. Nonetheless, it is highly likely that the first clinical impression contains many more signs and indicators. Therefore, the true value of the clinical impression has likely been underestimated by our analysis. Second, as this was a single-center study, its results may not be extrapolated to other centers, particularly not to other cultural settings [19]. Third, given that all 18 observations of the first clinical impression were collected by one researcher, we cannot exclude that recognition and interpretation of the first clinical impression are subject to clinically relevant inter-rater variability [20].

## 5. Conclusions

The first clinical impression of emergency patients presenting to the triage desk can predict the need for timely medical attention and hospital admission. Important features informing the prediction models were age, ability to walk, admission by emergency medical services, lying on a stretcher, breathing pattern, and bringing a suitcase. The inability to walk at triage presentation was highly predictive of both the need for timely medical attention and the need for hospital admission.

## Figures and Tables

**Table 1:** Characteristics of study patients.

*N*		1506
**Age**	*years*	60 (39–77)
**Male sex**	*n* (%)	731 (48.5)
**Mode of admission**		
*self-admission*	*n* (%)	820 (54.4)
*admission through EMS*	*n* (%)	668 (44.4)
*intra-hospital transfer*	*n* (%)	18 (1.2)
**Ability to walk at triage presentation**		
*able to walk unassisted*	*n* (%)	639 (42.4)
*walks with a limb*	*n* (%)	142 (9.4)
*can only walk with assistance (e.g., by another person or a walking aid)*	*n* (%)	26 (1.7)
*admitted in an EMS seat*	*n* (%)	529 (35.1)
*admitted on an EMS stretcher*	*n* (%)	170 (11.3)
**Body posture when seated for triage evaluation**	*n* (%)	1336 (88.7)
*no apparent abnormality*	*n* (%)	899 (59.7)
*stiff*	*n* (%)	101 (6.7)
*bent over*	*n* (%)	94 (6.2)
*hand on specific body part*	*n* (%)	73 (4.8)
*reduced muscle tone* *(in patients unable to walk)*	*n* (%)	55 (3.7)
*restless*	*n* (%)	16 (1.1)
**Body posture when lying on EMS stretcher**	*n* (%)	170 (11.3)
*supine*	*n* (%)	74 (4.9)
*semi-recumbent*	*n* (%)	68 (4.5)
*recovery position*	*n* (%)	14 (0.9)
*head of stretcher elevated > 60°*	*n* (%)	7 (0.5)
*supine with knee roll*	*n* (%)	7 (0.5)
**Sheets on stretcher unordered**	*n* (%)	21 (1.4)
**Facial expression**		
*no apparent abnormality*	*n* (%)	1061 (70.5)
*frowning*	*n* (%)	314 (20.8)
*anxious*	*n* (%)	64 (4.2)
*distorted in pain*	*n* (%)	60 (4)
**Skin color of the face**		
*normal*	*n* (%)	1153 (76.6)
*pale*	*n* (%)	223 (14.8)
*plethoric*	*n* (%)	90 (6)
*icteric*	*n* (%)	17 (1.1)
*cyanotic*	*n* (%)	13 (0.9)
*greyish*	*n* (%)	7 (0.5)
**Breathing pattern**		
*no apparent abnormality*	*n* (%)	1308 (86.9)
*increased work of breathing*	*n* (%)	107 (7.1)
*noisy, grunting breathing*	*n* (%)	57 (3.8)
*rapid breathing*	*n* (%)	29 (1.9)
**Mental impression**		
*normal*	*n* (%)	1137 (75.5)
*anxious*	*n* (%)	154 (10.2)
*disoriented*	*n* (%)	86 (5.7)
*fatigued*	*n* (%)	77 (5.1)
*depressed*	*n* (%)	31 (2.1)
*aggressive*	*n* (%)	10 (0.7)
*psychotic*	*n* (%)	4 (0.3)
**Vomitus bag present**	*n* (%)	31 (2.1)
**Clothes**		
*outdoor clothes*	*n* (%)	955 (63.4)
*indoor clothes*	*n* (%)	486 (32.3)
*night gown*	*n* (%)	52 (3.4)
*work clothes*	*n* (%)	14 (0.9)
**Hygienic state**		
*neat and groomed*	*n* (%)	1378 (91.5)
*unkempt*	*n* (%)	109 (7.2)
*urine odor*	*n* (%)	7 (0.5)
*alcohol odor*	*n* (%)	1 (0.1)
**Shoes**		
*street shoes*	*n* (%)	1238 (82.2)
*slippers*	*n* (%)	183 (12.2)
*no shoes*	*n* (%)	85 (5.6)
**Patient brought suitcase with him/her**	*n* (%)	418 (27.8)
**Chaperone present**	*n* (%)	373 (24.8)
**Conversation**		
*patient speaks with ED staff*	*n* (%)	852 (56.6)
*EMS staff speaks with ED staff*	*n* (%)	508 (33.7)
*chaperone speaks with ED staff*	*n* (%)	144 (9.6)
**Vital Parameters**		
*heart rate*	bpm	83 (73–98)
*systolic arterial blood pressure*	mmHg	145 (130–165)
*temperature*	°C	36.5 (36.3–36.8)
*plethysmographic oxygen saturation*	%	96 (95–98)
**Need for timely medical attention**	*n* (%)	275 (18.3)
**Need for hospital admission**	*n* (%)	571 (37.9)
**Need for intensive care unit admission**	*n* (%)	13 (0.9)
**In-hospital mortality**	*n* (%)	10 (0.7)

ED, emergency department; EMS, emergency medical services. Data are given as median values with interquartile ranges, if not otherwise indicated.

## Data Availability

The data presented in this study are available on request from the corresponding author. The data are not publicly available due to GDPR and local regulatory/data privacy provisions.
